# *Plasmodium knowlesi* and HIV co-infection in a German traveller to Thailand

**DOI:** 10.1186/1475-2875-12-283

**Published:** 2013-08-13

**Authors:** Jonas Ehrhardt, Andreas Trein, Peter Gottfried Kremsner, Matthias Frank

**Affiliations:** 1 Institute of Tropical Medicine, University of Tübingen, Wilhelmstr. 27, Tübingen 72074, Germany; 2 Group practice Schwabstraße 59, Schwabstraße 59, Stuttgart 70197, Germany

**Keywords:** *Plasmodium knowlesi*, Traveller, Thailand, *Plasmodium knowlesi* HIV co-infection, Viral load, Malaria, Malarone®, Severity

## Abstract

A case of *Plasmodium knowlesi* and HIV co-infection is reported in a German traveller returning from Thailand. The 54 year-old patient presented to the Institute of Tropical Medicine in Tübingen with a 11-day history of daily fever and chills. Initial microscopic evaluation of Giemsa-stained thin blood smears was suggestive of a mixed infection with *Plasmodium falciparum* and *Plasmodium malariae*. However, PCR amplification of small subunit ribosomal RNA gene revealed a *P. knowlesi* infection. Parasitaemia was 473 parasites/μl and the platelet count was within the normal range. Oral treatment with Malarone® was initiated and resulted in a fast recovery without any complications.

As part of routine screening the patient also underwent HIV testing and was found to be HIV positive with a CD4 cell count of 115/μl and a viral load of 34,799 copies/ml. A follow-up measurement of the viral load seven days after the first quantification revealed an increase to 102,000 copies/ml. Three months after the first quantification the viral load had dropped to 10,000 copies/ml without the initiation of antiretroviral treatment. This suggests the possibility of a *P. knowlesi* malaria-induced temporary elevation of viral load similar to that reported for *P*. *falciparum* and HIV co-infection.

## Background

In the last eight years a growing body of literature confirmed the existence of a fifth species of *Plasmodium* capable of causing human malaria. The fifth species is *Plasmodium knowlesi*, a malaria parasite naturally circulating in long-tailed (*Macaca fascicularis*) and pig-tailed (*Macaca nemestrina*) macaques in Southeast Asia. In 2004, Singh and colleagues described the first large focus of naturally acquired *P. knowlesi* infections in humans in the Kapit division of Sarawak (Malaysian Borneo) [1]. Thereafter, numerous reports followed and confirmed the existence of human *P. knowlesi* infections throughout Malaysian Borneo [2-5] and Peninsular Malaysia [2,6], as well as nearly every country in Southeast Asia (Myanmar [7], Vietnam [8], Philippines [9], Thailand [10], Cambodia [11], Singapore [12]). The frequency of *P. knowlesi* in patients with microscopically diagnosed malaria ranges from less than 1% (e g, in Thailand) [10] to up to 60% (Malaysian Borneo) [5]. Since 2004, twelve cases of imported *P. knowlesi* malaria to non-endemic countries have been published [13-24]. Eleven cases developed uncomplicated malaria and were successfully treated with common anti-malarial drugs, one patient improved spontaneously (without receiving treatment) [22]. However, lethal outcomes due to *P. knowlesi* malaria have been described in Southeast Asia. Case-fatality rates of 2% (district hospital) [25] and 11% (referral hospital) [26] have been reported. No deaths occured in a recent prospective study in a referral hospital in Malaysian Borneo, where severe *P. knowlesi* cases were treated with oral artesunate combination therapy or intravenous artesunate [27].

It has been reported that HIV infection increases the susceptibility to malaria [28,29] and the risk of developing severe *Plasmodium falciparum* malaria [30-33]. At the same time *P*. *falciparum* malaria causes a transient elevation of viral load in HIV-positive individuals [34-36]. The interactions between malaria and HIV have been studied mainly in African countries with *P*. *falciparum*-infected patients. There is currently no published information about *P. knowlesi* malaria and HIV co-infection available. This is the first description of *P. knowlesi* malaria in an HIV-positive adult with advanced immunosuppression.

## Case presentation

**Figure 1:**
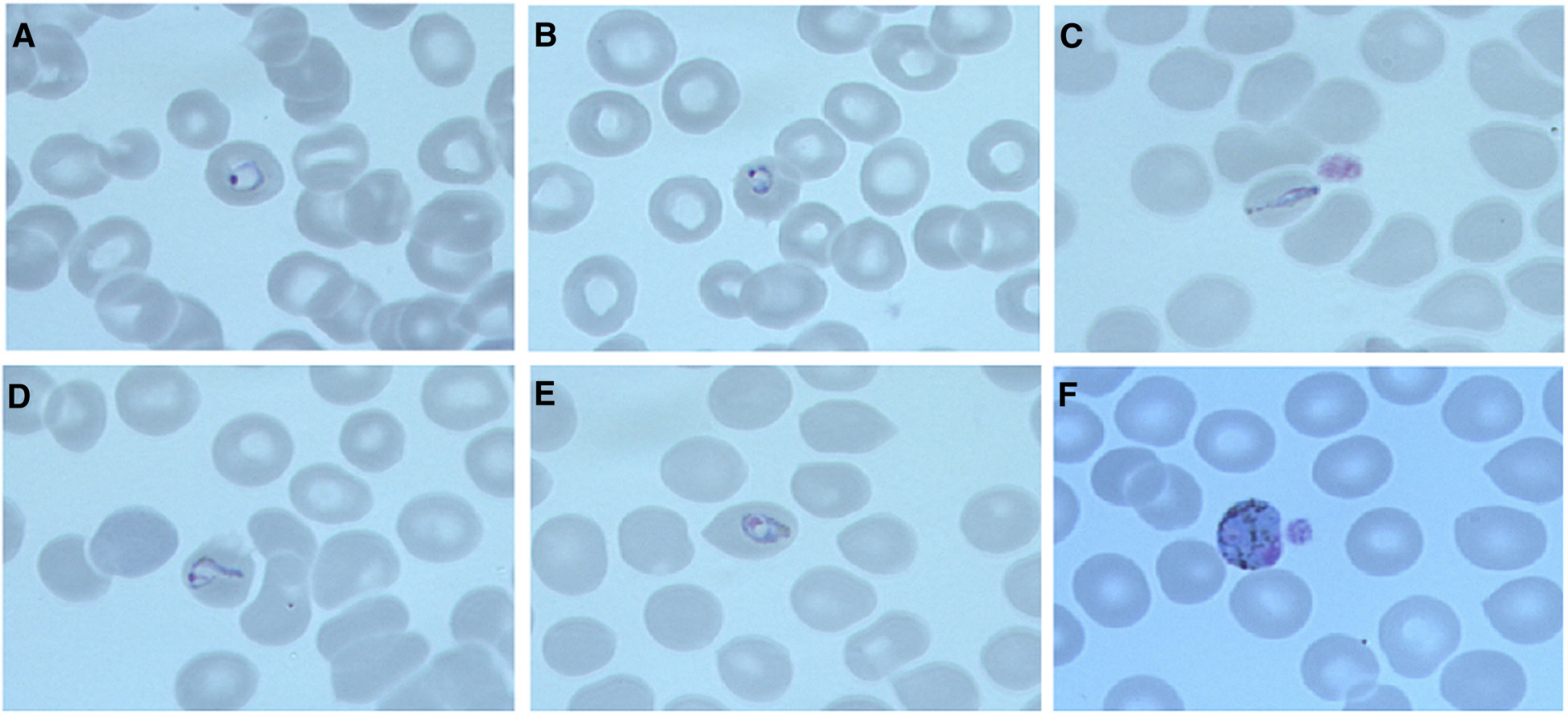
**Giemsa-stained thin blood smears with *Plasmodium knowlesi *parasites (magnification: 100×, oil immersion).** Panels **A** and **B** display ring stage parasites. Panel **C** shows a band-form. Panels **D** and **E** show late trophozoite parasites. Panel **F** displays a gametocyte.

A 54 year-old German traveller presented to the Institute of Tropical Medicine in Tübingen with fever, chills and severe headaches. Twenty days prior to presentation the patient had returned from a four-week trip to southern Thailand where he visited with his Thai wife. He stayed nearly all the time in Phuket and never took malaria chemoprophylaxis (please see below for a more detailed description of the trip). Eleven days prior to presentation the symptoms started with a sudden onset of fever (initially up to 40°C) and chills. Thereafter fever decreased gradually, but the patient continued to experience low grade fevers around 38.5°C until the day of presentation. Eight days prior to presentation the patient noticed a temporary rash on the lower limbs that he related to the intermittent use of Paracetamol. On examination, the patient was in no acute distress but appeared fatigued. The temperature was 37.4°C and vital signs were stable (blood pressure 120/75 mmHg, heart rate of 86 beats per minute). With the exception of tenderness in the left upper abdominal quadrant the physical examination was unremarkable. Abdominal ultrasound revealed mild splenomegaly (14.1 × 6.2 cm). Laboratory tests showed mild anaemia (haemoglobin 12.5 g/dl, normal 14–18 g/dl), elevation of lactate dehydrogenase (351 U/l, normal <251 U/l) and C-reactive protein (2.39 mg/dl, normal <0.51 mg/dl). Values were normal for platelet count (197,000/μl, normal 150,000 – 400,000/μl), white cell count (4.100/μl, normal 4,000 – 9,500/μl), total bilirubin (0.8 mg/dl, normal <1,2 mg/dl) and plasma creatinine (0.9 mg/dl, normal 0.6 – 1.1 mg/dl). Rapid diagnostic tests for dengue were negative (Dengue Dx IgG/IgM and NS1 Antigen Rapid Test, Focus Diagnostics, USA). Giemsa-stained thick and thin blood smears showed the presence of malaria parasites (Figure 1). Using the Lambaréné method parasitaemia was estimated to be about 0.01% (473 parasites/μl). Microscopic evaluation of thin blood smears revealed several ring stage parasites with a morphology reminiscent of *P*. *falciparum* ring stage parasites. However, several late trophozoite stage parasites with haemozoin pigment as well as gametocytes with a size of an uninfected red blood cell were identified. Based on these morphologic features the presumptive diagnosis of a co-infection with *P*. *falciparum* and *Plasmodium malariae* was made*.* Treatment with atovaquone/proguanil 250 mg/100 mg (four tablets/day for three days) was initiated and the patient was admitted to the hospital. During anti-malarial therapy the parasitaemia cleared rapidly. Serial blood smears revealed a parasitaemia of 70/μl after 24 hours and 11/μl after 48 hours of therapy. The platelet count after 24 hours and 48 hours of therapy was 249.000/μl and 257.000 μl respectively.

Due to the uncertain blood smear identification of *Plasmodium* species, small subunit ribosomal RNA gene PCR amplification was performed. The PCR fragment was sequenced and a BLAST search in the GenBank database (basic local alignment search tool) showed a 96%-match with *P. knowlesi* sequences. No other human plasmodial species were identified by the BLAST search. The patient was discharged after three days in improved conditions.

In addition, at day of presentation HIV testing was performed as part of routine screening. The patient’s last testing dated four years back and had been negative. Now HIV-1-antibodies were detected by ELISA and confirmed by Western blot. The CD4 cell count was 115/μl and viral load was 34,799 copies/ml. On repeated questioning the patient reported that the relationship with his Thai wife started four years ago. At that point both partners underwent HIV testing and were found to be HIV negative. During the patient’s yearly visits to Thailand the couple had unprotected intercourse. The patient stated that except for his wife he had not had any other sexual partners for the last four years. An illness suggestive of acute HIV could not be recalled.

Seven days after the day of presentation the viral load measurement was repeated at an outside physician’s office and showed an increase to 102,000 copies/ml. Eleven days after the day of presentation the patient presented for a follow-up examination. The physical examination was without pertinent positive findings. However, the patient continued to complain about fatigue and night sweats. A repeat blood smear was negative for malarial parasites. The complete blood count revealed a white blood cell count of 3,200 cells/μl and unchanged haemoglobin of 12.2 g/dl. The values for lactate dehydrogenase and C-reactive protein were within normal limits. More detailed questioning of the travel history revealed that the patient had stayed three weeks at his house in Phuket where he lived with his wife. From the last week of the four-week trip he had spend three days in Ranong Province (southern part of the border to Myanmar). He noted that while walking in the local forests and fishing in the Andaman Sea he suffered a lot of mosquito bites. There was no contact with monkeys reported. For long-term HIV care the patient was referred to a specialized HIV centre. Five weeks after the *P. knowlesi* infection the viral load follow-up revealed a drop to 17,000 copies/ml without antiretroviral therapy. Three months after the *P. knowlesi* infection, viral load was 10,000 copies/ml still without antiretroviral therapy. The CD4 cell count was 204/μl and 264/μl respectively (Table 1).

**Table 1:** Development of the viral load and CD4 count

	Day 1*	**Day 7**	**Day 37**	**Day 87**
			**(week 5)**	**(week 12)**
Viral load (copies/μl)	34 799	102 000	17 000	10 000
CD4 count (cells/μl)	115	223	204	264

*Day of presentation of the patient with acute *P. knowlesi* malaria.

According to official guidelines [37], the patient met criteria for the initiation of antiretroviral therapy. The necessity of starting antiretroviral therapy was repeatedly discussed with the patient. However, he repeatedly rejected this due to his personal attitude towards conventional medicine and instead expressed hope of spontaneous improvement.

## Conclusions

*Plasmodium knowlesi* cases have been detected in several regions of Thailand mainly in forestal areas bordering Cambodia in the east, Malaysia in the south and Myanmar in the north-west [10]. A recent study confirmed the transmission of *P. knowlesi* in Ranong Province (southern part of the border to Myanmar) [38], where the here presented patient stayed for three days and where he most likely acquired the infection. The overall incidence of *P. knowlesi* malaria in Thailand is low. Among 1874 febrile patients attending malaria clinics in different areas of Thailand the overall prevalence of *P. knowlesi* was 0.57% [10]. Nevertheless, this is already the second imported case of a travel-related *P. knowlesi* infection acquired in Thailand [21]. This indicates that even short-term travellers are at risk of *P. knowlesi* infections and that due to increasing travel activities to Southeast Asia, imported *P. knowlesi* malaria might become more important in non-endemic countries.

During the initial questioning the patient stated that he had stayed all the time of the voyage in Phuket where *Plasmodium* species transmission is minimal [39] and no cases of *P. knowlesi* infections have been reported. Only on repeat questioning during the follow-up visit did the patient recall a short visit to Ranong Province. This serves as a reminder that malaria has to be excluded in returning travellers from Thailand, even if they report primarily visiting areas with very low incidence of malaria.

It has been shown for *P. falciparum* malaria that HIV-positive individuals have a temporarily increased viral load during acute malaria [34-36]. Kublin and colleagues described an increase of viral load during symptomatic malaria (median log increase of 0.42, p < 0.0001) and a subsequent decrease of viral load back to baseline eight weeks after anti-malarial treatment [34]. A similar observation could be made in the here presented HIV-positive adult with acute *P. knowlesi* malaria. A baseline measurement before the acute malaria infection was not available as the HIV infection was diagnosed for the first time. Nevertheless, an increase in viral load was observed shortly after the *P. knowlesi* infection with a subsequent drop at follow-up examinations five and 12 weeks later without the initiation of antiretroviral therapy. These findings suggest a possible influence of acute *P. knowlesi* malaria on pre-existing HIV infection, resulting in a temporary increase in viral load as it has been described for acute *P. falciparum* malaria. However, as this is an observation in one patient it is necessary to assess more cases in order to confirm this finding.

The described finding raises the question as to whether there are other similarities between clinical characteristics of HIV-*P. falciparum* co-infection and those of HIV-*P. knowlesi* co-infection. Most *P. knowlesi*-infected individuals develop clinically uncomplicated malaria, but severe cases with lethal outcomes have been described recently [2]. It has been shown for *P. falciparum* malaria that HIV-positivity is a risk factor for the development of severe malaria and death [30-33]. In a prospective cohort study in South Africa, HIV-infected individuals with a CD4 cell count <200/μl had an increased risk of severe malaria (odds ratio 4.8) [30]. Two studies investigating imported *P. falciparum* malaria to non-endemic countries showed that severe malaria was more frequent in patients with a CD4 cell count <350/μl, (odds ratio 2.5 and 3.2) [32,33].

The clinical course of the *P. knowlesi* malaria case described here was uncomplicated despite marked immunosuppression of the patient (115 CD4 positive cells/μl). Two research groups recently established the hight of parasitaemia as a good predictor for the risk of severe disease (≥35,000 parasites/μl [40] and >20,000 parasites/μl [27]). The parasitaemia observed in the here presented patient was relatively low (473 parasites/μl). Therefore the uncomplicated course of the patients’ disease supports the apparent correlation of height of parasitaemia and severity of disease in *P. knowlesi* malaria. Anaemia was mild and there was neither acidosis nor renal failure, which are frequently associated with severe *P. knowlesi* infections [26,40].

Interestingly, thrombocytopaenia, which is nearly universal in patients with *P. knowlesi* infection [25-27], was not present. However, it is important to note that the platelet count was at the lower limit of the normal range on presentation and increased after the beginning of antimalarial therapy. It has recently been published that thrombocytopaenia in *P. knowlesi* malaria improves on average 24 hours after successful parasite clearance, suggesting a possible relation between *P. knowlesi* parasitaemia and a decreased platelet count [27]. It is therefore possible that the low parasitaemia of the described patient resulted in a relative small decrease of the platelet count. In addition the nadir of the platelet count may have occurred prior to presentation since the patient only presented eleven days after the onset of symptoms. Previous *P. knowlesi* cases that are published without the development of thrombocytopaenia include a 11-year old boy with chronic myeloid leukaemia taking imatinib [5] and two asplenic patients [27]. Taken together those cases suggest a possible immune-related component in the development of thrombocytopaenia during acute *P. knowlesi* malaria. It is tempting to speculate that the HIV co-infection contributed to the absence of thrombocytopaenia in the presented case, however it is not possible to address this question on the basis of a single case report.

The current case is the first description of *P. knowlesi* malaria in an HIV-positive adult with advanced immunosuppression. The patient experienced a temporary elevation of the viral load that might have been due to the acute *P. knowlesi* malaria. Despite advanced immunosuppression the patient’s laboratory parameters were not suggestive of a severe *P. knowlesi* infection and he had an uncomplicated clinical course.

## Consent

Written informed consent was obtained from the patient for publication of this case report and any accompanying images. A copy of the written consent is available for review by the Editor-in-Chief of this journal.

## Abbreviations

BLAST: Basic local alignment search tool; CD: Cluster of differentiation; HIV: Human immunodeficiency virus; PCR: Polymerase chain reaction.

## Competing interests

The authors declare that they have no competing interests.

## Authors’ contributions

JE reviewed the literature, conducted the collection and interpretation of data and drafted the manuscript. AT helped with the data collection and data interpretation and revised the manuscript. PK provided conceptual advice. MF revised the manuscript, helped to interpret data, helped to draft the manuscript and has given final approval of the version to be published. All authors read and approved the final manuscript.
